# Supplementary material for: The WW domain of IQGAP1 binds directly to the p110α catalytic subunit of PI 3-kinase
Source: Biochem J. Author manuscript; Available in PMC 2024 Nov 5. (PMC10625650; doi:10.1042/BCJ20220493)
Supplement: Supplementary Material [file NIHMS1930033-supplement-Supplementary_Material.pdf]

Supplementary Materials

The WW domain of IQGAP1 binds directly to the p110α catalytic subunit of PI 3-kinase

by A.J. Bardwell *et al.*

Supplementary Figures

1. Specificity control: p110α does not bind to the N-terminal domains of MEK1 or MEK2
2. The WW domain of IQGAP binds to p110α/p85α and p110α/p65α heterodimers.
3. Alignment of 94 human WW domains.
4. Sequence tree of 94 human WW domains.
5. Human WW domains most closely related to the WW domain in human IQGAP1.
6. Sequence logos of human WW domains and of vertebrate IQGAP1 orthologs.

Supplementary Tables

1. Binding assay data for WW-p110α interaction.
2. PCR primers used in this study.

References

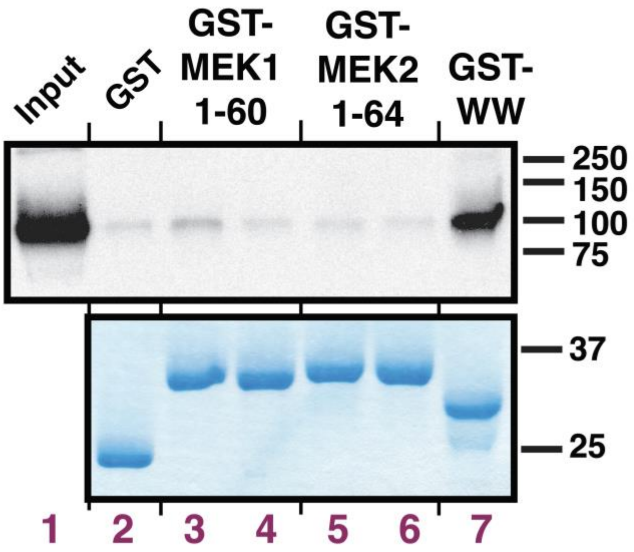

Supplementary Figure 1. Specificity control: p110α does not bind to the N-terminal domains of MEK1 or MEK2.

^{35}S-radiolabeled full-length human p110α protein was prepared by *in vitro* translation and partially purified by ammonium sulfate precipitation, and a portion (5% of the amount added in the binding reactions) was resolved on a 12% SDS-polyacrylamide (SDS-PAGE) gel (lane 1). Portions (~1 pmol) of the same protein were incubated with 25 μg of the indicated GST fusion proteins bound to glutathione-Sepharose beads (lane 2-7), and the resulting bead-bound protein complexes were isolated by sedimentation and resolved by 12% SDS-PAGE on the same gel. The gel was analyzed by staining with a Coomassie-blue-based reagent for visualization of the bound GST fusion protein (lower panel) and by X-ray film exposure for visualization of the bound radiolabeled protein (upper panel). The migration of molecular weight markers is indicated on the right.

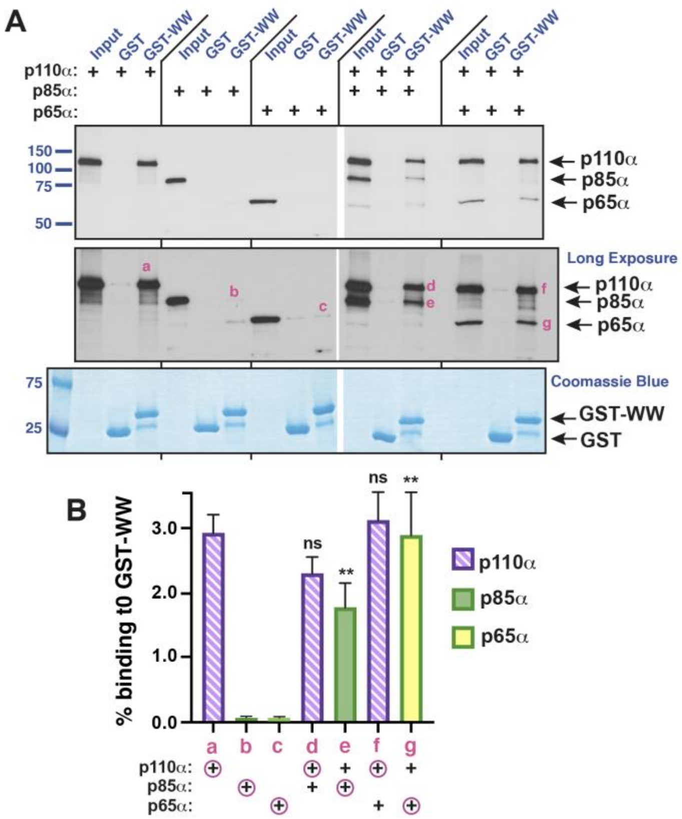

Supplementary Figure 2. The WW domain of IQGAP binds to p110α/p85α and p110α/p65α heterodimers.

(**A**) Human p110α, p85α, and p65α were produced by coupled *in vitro*-transcription and translation (TNT) in separate reactions. In addition, p110α + p85α were both translated in the same reaction, and p110α + p65α were both translated in the same reaction. The products of these 5 TNT reactions were then each tested for binding to GST or GST-WW. The figure shows an autoradiogram of a representative experiment (the experiment is different from that shown in Figure 4B and 4D). Top panel, standard exposure; middle panel, longer exposure; bottom panel, Coomassie-blue-based staining to visualize GST and GST-WW. Other details as in Figure 1. The pink lower case letters a-g in the middle panel indicate bands representative of those quantified in **B**.

(**B**) Quantification of the binding of human p110α, p85α and p65α to GST-WW. Data are an average of 6-10 independent repetitions of the binding assay described above and depicted in Figure 4A, measured as percent of input bound. The lowercase letters a-g below the graph match up with bands labeled in the middle panel of **A**: 'a' is p110α alone binding to GST-WW, 'd' is p110α binding to GST-WW when co-TNT'd with p85α, etc. The circled '+' signs indicate which protein band is being quantified in the bar graph above. GST alone backgrounds have been subtracted from all points. Error bars show the standard error of the mean. Significance estimates are shown for the following pairwise comparisons: d to a, e to b, f to a, and g to c. **, $p < 0.01$; ns, not significant ($p > 0.05$).

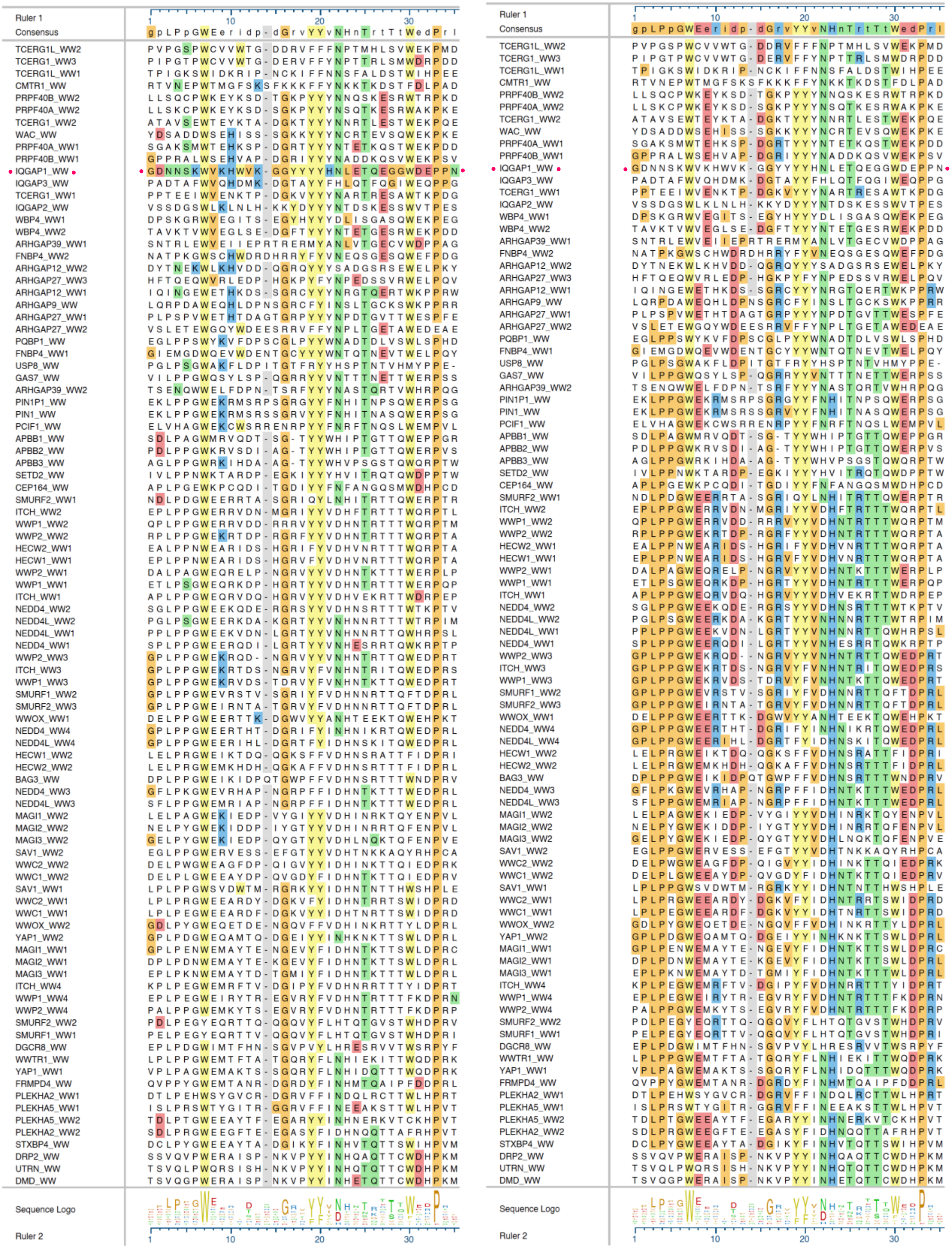

Supplementary Figure 3. Alignment of 94 human WW domains.

Supplementary Figure 3. Alignment of 94 human WW domains.

The sequences are listed in the order shown in the Tree in Supplementary Figure 4. The left and right alignments are identical except for coloring. On the left, residues are colored if they match IQGAP1; on the right, residues are colored if they match the consensus shown on top. The color scheme is as follows: aromatic residues (F, Y, W) are yellow, acidic residues (D, E) are red, nonpolar residues (A, G, I, L, M, P, V) are orange, polar residues (C, N, Q, S, T) are green. The numbering on the rulers are different than the numbering in Figures 6A and 7A. To convert, subtract 6 from the ruler numbers to the left of the gap in the IQGAP1 sequence and subtract 7 from the ruler numbers to the right of the gap. The alignment was generated using MegAlign Pro version 17.3.1 (DNASTAR, Inc.) using the MAFFT L-ins-I algorithm with a gap open penalty of 3 and a gap extension penalty of 0. BLOSUM62 was used as the scoring matrix.

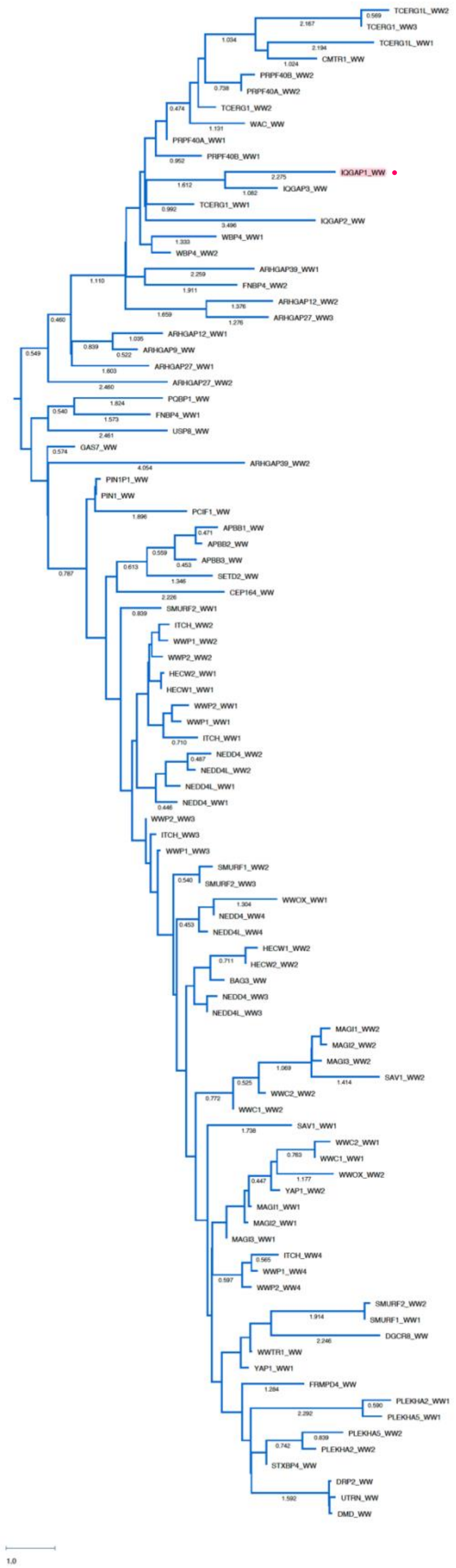

Supplementary Figure 4.
Sequence tree of 94 human WW domains.
Sequence identity tree generated from the alignment shown in Supplementary Figure 3, using the maximum likelihood method with uncorrected pairwise distance and pairwise gap removal options. Other details as in Supplementary Figure 3.

Sort by Similarity		Sort by Identity		Sort by Score	
Designation	Similarity	Designation	Identity	Designation	Score
IQGAP1_WW	100.0	IQGAP1_WW	100.0	IQGAP1_WW	213
IQGAP3_WW	64.7	IQGAP3_WW	38.2	IQGAP3_WW	77
WBP4_WW2	55.9	FNBP4_WW1	35.3	PRPF40A_WW1	68
PRPF40A_WW1	52.9	ARHGAP12_WW1	35.3	WBP4_WW2	62
IQGAP2_WW	52.9	WBP4_WW2	32.4	WAC_WW	60
WBP4_WW1	52.9	PRPF40A_WW1	32.4	WBP4_WW1	59
PRPF40B_WW1	50.0	WWP2_WW3	30.3	TCERG1_WW1	58
FNBP4_WW1	47.1	TCERG1_WW2	29.4	PRPF40B_WW1	57
TCERG1_WW2	47.1	WAC_WW	29.4	FNBP4_WW1	55
WAC_WW	47.1	ARHGAP12_WW2	29.4	TCERG1_WW2	55
PRPF40A_WW2	47.1	TCERG1_WW1	29.4	ARHGAP12_WW2	54
PRPF40B_WW2	47.1	ARHGAP39_WW1	29.4	IQGAP2_WW	53
CMTR1_WW	47.1	DMD_WW	29.4	ARHGAP12_WW1	51
ARHGAP27_WW2	47.1	IQGAP2_WW	26.5	PRPF40A_WW2	45
ARHGAP12_WW2	44.1	WBP4_WW1	26.5	WWOX_WW1	43
TCERG1_WW1	44.1	PRPF40B_WW1	26.5	ARHGAP39_WW2	42
APBB1_WW	42.4	PRPF40A_WW2	26.5	FNBP4_WW2	40
ARHGAP12_WW1	41.2	FNBP4_WW2	26.5	APBB1_WW	39
FNBP4_WW2	41.2	ITCH_WW3	26.5	ITCH_WW3	39
ITCH_WW3	41.2	PCIF1_WW	26.5	PRPF40B_WW2	39
PCIF1_WW	41.2	ARHGAP27_WW1	26.5	WWP2_WW3	36
WWOX_WW1	41.2	GAS7_WW	26.5	DMD_WW	35

Supplementary Figure 5. Human WW domains most closely related to the WW domain in human IQGAP1.

Relatedness to IQGAP1's WW domain was assessed by three methods: sequence similarity (left columns), sequence identity (middle columns), and the score generated by a Needleman-Wunsch global pairwise alignment with a gap penalty of 10, a gap extension penalty of 10, and BLOSUM62 used as the scoring matrix. The top 25 in each category are shown. Select WW domains are color coded as a visual aid. The sequences used are the same as shown in Supplementary Figure 3.

Human WW domains

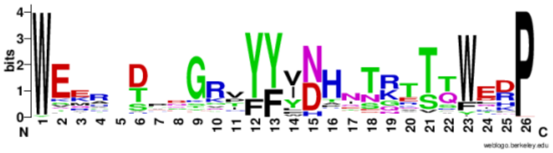

Mammalian IQGAP1:

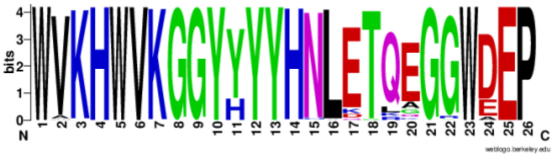

Bird IQGAP1:

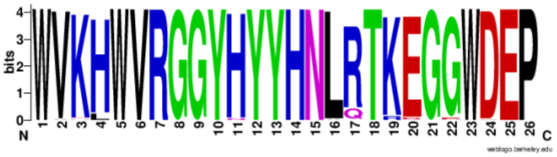

Reptile IQGAP1:

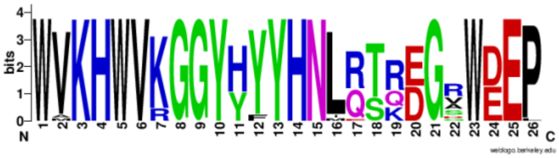

Amphibian IQGAP1:

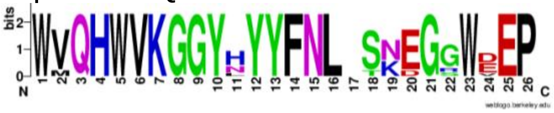

Bony Fish IQGAP1:

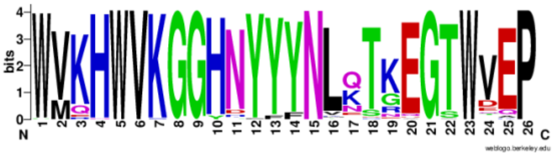

Other Fish IQGAP1:

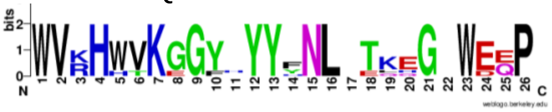

Supplementary Figure 6. Sequence logos of human WW domains and of vertebrate IQGAP1 orthologs.

The sequence logos were created with WebLogo [1]. Residue numbering as in Figures 6A and 7A. Vertebrate ortholog sequences used to create the logos are from the National Center for Biotechnology Information (NCBI) page on IQGAP1 orthologs: https://www.ncbi.nlm.nih.gov/gene/8826/ortholog/?scope=89593.

Supplementary Table 1. Binding assay data for WW-p110α interaction.

Experiment [a]	Binding % [b]	K_d, μM [c]
1	5.1	73
2	5.0	75
3	4.9	76
4	4.9	76
5	4.7	80
6	4.5	83
7	4.1	92
8	3.8	100
9	3.6	105
10	3.6	105
11	3.4	112
12	3.4	112
13	3.1	123
14	2.7	142
15	2.5	153
16	2.2	175
17	2.1	183
18	2.0	193
19	1.9	203
20	1.7	227
21	1.7	227
**Mean**	**3.4**	**129**
**Median**	**3.4**	**112**
**Standard Deviation**	**1.2**	**52**
**Standard Error**	**0.26**	**11**
**95% Confidence Interval**	**2.8-3.9**	**105-153**

[a]Binding reactions (200 μl) contained ~ 1 pmole (~ 5 nM) ^{35}S-labeled, *in vitro*-translated, full-length p110α protein and 25 μg (3.9 μM) GST-WW fusion protein. The experiments are sorted by observed K_d from lowest to highest.
[b]Percent of the input ^{35}S-labeled protein which bound to the GST fusion protein.
[c]Dissociation constant (K_d), in μM, calculated based on the known input concentrations and percent binding, as described elsewhere [2, 3]. Lower K_d's indicate tighter binding.

Supplementary Table 2. PCR primers used in this study.

Name	Sequence (5'→3')	Use
hIQGAP1(679-x)	ggaggcggtGGATCCacc**ATG**ggagataataacagcaagtgg	GST-WW
hIQGAP1(x-719)	gccgctcgaGTCGAC**TTA**ctgcatagaattttgcacaaaa	GST-WW
hIQGAP1(743-x)	agctcggtaCCCGGGacc**ATG**ctggccaatgaaggcctga	IQGAP1(743-1657)
hIQGAP1(x-1657)	tgcctgcagGTCGACtggcagcaaacgatcaa**TTA**	IQGAP1(743-1657)
p110-1-x	gcgGGATCCacc**ATG**cctccaagaccatcatca	p110α(1-1068)<br>p110α(1-190)
p110-end-down	cgGTCGAC**TTA**gttcaaagcatgctgctt	p110α(x-1068)
p110-6-x	gcg**GGATCC**accATGtcatcaggtgaactgtgg	p110α(6-123)
p110-x-123	cgGTCGAC**TTA**catgccgatagcaaaacc	p110α(6-123)
p110-x-190	cgGTCGAC**TTA**tatttgccctttatccaa	p110α(1-190)
p85up	gcgGGATCCacc**ATG**agtgctgaggggtaccag	p85α(1-724)
p85down	cgGTCGAC**TTA**tcatcgcctctgctgtgcata	p85α(1-724)
GST-WW-up	gcGAATTCacc**ATG**tcccctatactaggttat	pcDNA-GST, etc.
GST-WW-down	cgcTCTAGAccgaaacgcgcgaggcagatc	pcDNA-GST, etc.

Introduced restriction sites are in uppercase text; start and stop codons are in bold text.

References for Supplementary Materials

1 Crooks, G. E., Hon, G., Chandonia, J. M. and Brenner, S. E. (2004) WebLogo: a sequence logo generator. Genome Res. **14**, 1188-1190

2 Bardwell, A. J., Wu, B., Sarin, K. Y., Waterman, M. L., Atwood, S. X. and Bardwell, L. (2022) ERK2 MAP kinase regulates SUFU binding by multisite phosphorylation of GLI1. Life Sci Alliance. **5**

3 Bardwell, L. and Shah, K. (2006) Analysis of mitogen-activated protein kinase activation and interactions with regulators and substrates. Methods. **40**, 213-223
